# Heritability of telomere variation: it is all about the environment!

**DOI:** 10.1098/rstb.2016.0450

**Published:** 2018-01-15

**Authors:** Hannah L. Dugdale, David S. Richardson

**Affiliations:** 1 Faculty of Biological Sciences, School of Biology, University of Leeds, Leeds LS2 9JT, UK; 2 School of Biological Sciences, University of East Anglia, Norwich Research Park, Norwich, Norfolk NR4 7TJ, UK

**Keywords:** heritability, telomeres, environmental effects, genetic effects, animal models, variation

## Abstract

Individual differences in telomere length have been linked to survival and senescence. Understanding the heritability of telomere length can provide important insight into individual differences and facilitate our understanding of the evolution of telomeres. However, to gain accurate and meaningful estimates of telomere heritability it is vital that the impact of the environment, and how this may vary, is understood and accounted for. The aim of this review is to raise awareness of this important, but much under-appreciated point. We outline the factors known to impact telomere length and discuss the fact that telomere length is a trait that changes with age. We highlight statistical methods that can separate genetic from environmental effects and control for confounding variables. We then review how well previous studies in vertebrate populations including humans have taken these factors into account. We argue that studies to date either use methodological techniques that confound environmental and genetic effects, or use appropriate methods but lack sufficient power to fully separate these components. We discuss potential solutions. We conclude that we need larger studies, which also span longer time periods, to account for changing environmental effects, if we are to determine meaningful estimates of the genetic component of telomere length.

This article is part of the theme issue ‘Understanding diversity in telomere dynamics'.

## 1. Introduction

**Figure 1.**
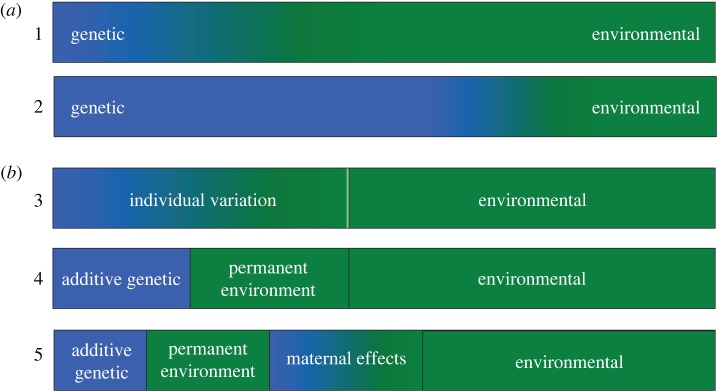
Hypothetical proportion of telomere length variation among individuals explained by genetic (blue) and environmental (green) effects: (*a*) in a population where individuals experience: (1) highly variable environments versus (2) relatively constant environments; and (*b*) estimated using mixed models of increasing complexity, based on repeated measures of telomere length per individual: model (3) a mixed model to separate individual variation from environmental (residual) variation, model (4) an ‘animal’ model where individual variation is separated into additive genetic and permanent environmental effects, and model (5) where maternal identity is included to estimate maternal effects.

Phenotypic variation is the result of both genetic and environmental effects. To understand the causes and consequences of variation in any given trait—as we must to fully appreciate its ecological, evolutionary and health implications—we need to determine the contribution of these two components. This is difficult as environmental and genetic effects are complex and intertwined; they include many interacting aspects, e.g. additive, dominant or epistatic genetic effects, and environmental effects that may be variable or constant [1,2]. Quantitative genetics offers an analytical framework to investigate the causes and evolutionary consequences of phenotypic variation, particularly the genetic component. However, it is important to understand that in quantitative genetic analyses we are often measuring relative effects. For example, when determining the heritability of a trait, we are estimating the contribution of genetic effects to the total phenotypic variance observed in that trait within a population (figure 1*a*). Any change in the influence of the environment upon that trait will alter our estimate of heritability, even when there is no change in the underlying genetic variation. It is, therefore, important to fully understand and take into account environmental effects in any quantitative genetic study [1].

Telomere length (or dynamics) is a phenotypic trait influenced by genetic and environmental effects [3,4]. The basic function of telomeres is to act as a chromosomal cap and maintain the integrity of linear chromosomal DNA [5]. Initial telomere length is inherited [5], but telomeres typically shorten with age in somatic cells due to the DNA end replication problem during cell division [6] and other factors, most notably damage caused by oxidative stress [7,8]. Oxidative stress is elevated by many environmental factors [7,9] and, as a result, is thought to be why telomere attrition is accelerated by the different stresses experienced during an individual's life [10,11]. Telomere restoration can also occur due to mechanisms such as telomerase activity [12] but telomerase is thought to be downregulated in the somatic cells of many adult organisms [13]. Importantly, critically short telomeres induce cell senescence or death [14,15] and the accumulation of such cells over time has been linked to organismal senescence [14,16]. Telomere length, or rate of attrition, has now been linked to lifespan among species [17,18], and to survival probability and lifespan within many species [19–22], though the causality of this association remains unclear [23,24]. Consequently, understanding which factors determine variation in telomeres is of considerable importance.

From a quantitative genetics perspective, the goal is to determine the contribution of genetic effects to among-individual variation in telomeres. Only by having accurate estimates of the genetic component of telomere variation can we determine its evolutionary potential [25]. However, individual telomere length at any given point is dependent on three processes: the initial length of the zygote's telomeres, the amount of attrition experienced and the amount of restoration. These processes may all be influenced by both genetic and environmental factors [4,26] and their relative contribution will differ among individuals and change throughout an organism's life. If we are to measure genetic effects accurately we need to ensure that environmental influences are carefully controlled for, either physically or statistically. Laboratory studies can reduce or isolate environmental variation. Such studies provide an excellent way to investigate how specific environmental factors influence telomere length, and provide important insights (e.g. [10,11]). However, if environmental variation is minimized then, by definition, the majority of phenotypic variation will be due to genetic effects and the heritability of the trait will approach 1 (figure 1*a*). Knowing the heritable component under such conditions is not, in itself, that useful. We need to be able to determine the relative contribution of genetic effects to variation in telomere length, under the conditions in which organisms live naturally, if we are to understand its consequences in terms of their health, ecology and evolution.

Determining when the environment is accelerating telomere attrition is also important in its own right [27,28]. For example, measuring effects linked to habitat quality, early-life environments or captive conditions [10,20,29,30] will provide insight into medical, veterinary, conservation and ecological issues. Such studies can be especially revealing if they allow us to measure chronic effects not detectable through immediate patterns of mortality or body condition, but which have long-term consequences, e.g. pathogen infection [31], stress [11] and environmentally dependent inbreeding [32]. Furthermore, the amount of telomere shortening caused by different environmental effects could provide researchers with a generic currency with which to measure the relative impact of different environmental stressors [30] and thus gain insight into the trade-offs that occur throughout an individual's life [24].

The importance of estimating environmental effects in any quantitative genetics study of telomeres is, therefore, clear. In the rest of the paper, we will first outline two key complications: (i) how environmental effects (and thus estimates of heritability) change over space and time, and (ii) the importance of recognizing that telomeres are not a fixed trait, but can change extensively with age. We then outline specific sources of variation that may impact telomeres, before discussing how these can be included in analyses, what has been analysed to date in vertebrate studies, what problems exist in those studies and finally, how the field can best move forward.

## 2. Genetic and environmental factors contribute to variance in telomere length

Complex phenotypic traits, such as telomere length, are rarely underpinned by a few genes of large effect; rather, they are primarily a result of the action of many genes of small effect [33]. At each locus, effects may be due to additive or dominance effects. Different genes will also impact the resulting phenotype in different ways, i.e. with additive or epistatic actions. These genetic effects could alter telomere length in various ways, such as: initial telomere length in the fertilized egg, individual resistance to telomere attrition or the extent of telomerase expression. Quantitative genetics does not require knowledge of the genes underlying telomere length, or the way in which the genes act. Rather, it assumes that phenotypic traits result from many genes which each have an infinitesimally small, additive effect on the phenotype [34,35]. From an evolutionary perspective, additive genetic variance is of particular interest as it is used to calculate the heritability of the trait, which in combination with the strength of selection on the trait will determine its evolutionary potential [34].

From an environmental perspective, telomeres may be impacted by effects from a wide variety of sources (e.g. natal, population, parental) that may differ in type (e.g. variable, constant). Environmental effects can include both common environmental effects, i.e. that affect a group of individuals experiencing the same environment, and permanent environmental effects, i.e. that have a consistent effect over an individual's lifetime [36]. Population-wide cohort effects [20,32,37] could thus represent common environment effects on individuals from a particular cohort, or permanent environment effects if the effect on a particular cohort lasts over lifetimes. Environmental effects can also include parental effects. Parental effects on telomere length [38] could arise for several reasons, such as: epigenetic effects (e.g. DNA methylation), differential contributions to an egg, parental care effects or as a direct result of local physical conditions provided by the parents. Confusingly, while parental effects act through the environment provided to offspring, they can have a genetic component. For example, provisioning variation between parents can impact offspring telomeres [39] either as a result of environmental effects, e.g. higher food abundance in good territories, or genetically determined differences in parental provisioning behaviour [40].

## 3. Environmental effects change over space and time

**Figure 2.**
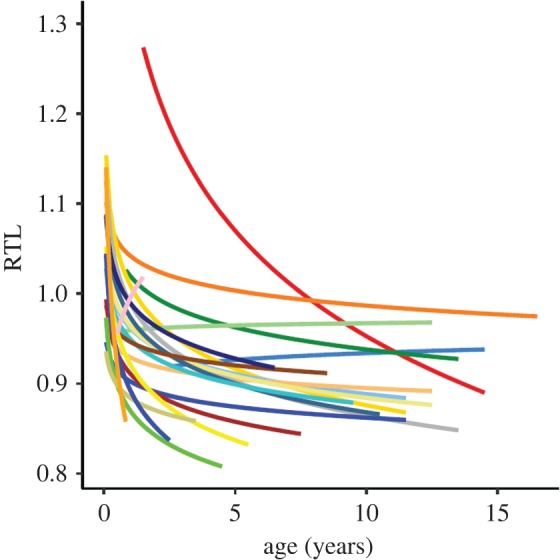
Relative telomere length (RTL) among cohorts in relation to age in Seychelles warblers, *Acrocephalus sechellensis*. Lines represent fitted values from a linear regression of RTL and log-transformed age. Colours represent birth years (1993–2014). Adapted from [30].

Under natural conditions the environment, and thus its impact on telomeres, may change considerably, both spatially and temporally within a population. The best evidence of this is provided by long-term ecological studies. For example, in Soay sheep, *Ovis aries* [20] and Seychelles warblers, *Acrocephalus sechellensis* [30] varying environmental conditions during early life generate considerable and long-lasting cohort effects on telomere length (figure 2). The effect of such spatio-temporal variation on quantitative genetic studies of telomere dynamics needs to be considered carefully. If the impact of the environment changes, then the relative amount of telomere variation due to genetic versus environmental effects will differ. This is not error but a real effect we need to understand. To add to this complexity, genotype × environment (G × E) interactions may occur [41]. For example, certain genetic effects may only be apparent under stressful conditions. Indeed, a recent study found evidence that lower individual heterozygosity due to inbreeding resulted in faster telomere attrition, but only under poor environmental conditions [32]. Given the above, any estimates of genetic effects on telomere length in natural populations will depend on when and where the study takes place. With this in mind, and taking into account that studies are normally very restricted on a spatial and temporal scale, it may not be surprising if different studies, even on the same species (as seen in humans; table 1), vary greatly in their estimates of genetic effects [56].

**Table 1.** Summary of studies estimating narrow-sense heritability (*h*^2^) of telomere length (TL) in vertebrates. qPCR, quantitative polymerase chain reaction; TRF, telomere restriction fragments; n.s., not significant; MZ, monozygotic twins; DZ, dizygotic twins.

ref.	species	method	Parent and offspring age at sampling controlled for?	Parental age at conception controlled for?	Environment controlled for?	statistics	*N*^a^	*h*^2^ (95% CI)^d^
[42]	human *Homo sapiens*	Southern blot	yes: twins sampled at 4, 17 and 44 years	no	yes: shared environment	MZ twins DZ twins	59 56	0.78 (0.69–0.87) 0.78 (0.69–0.87)
[43]	human *Homo sapiens*	Southern blot	yes: age as a covariate	no	no	linear mixed model (twin data)	47	0.84
[44]	human *Homo sapiens*	Southern blot	yes: age-adjusted telomere length	no	telomere length adjusted for smoking	father–son father–daughter mother–son mother–daughter sister–sister sister–brother brother–brother	34 47 51 71 22 25 23	n.s. 1.20 0.82 1.18 1.22 1.42 1.66
[45]	human *Homo sapiens*	Southern blot	no	no	no	MZ twins (73–79 yr) DZ twins (73–79 yr) MZ twins (80–94 yr) DZ twins (80–94 yr) MZ twins (73–94 yr) DZ twins (73–94 yr)	89 114 39 45 128 159	0.31 0.54 0.34 n.s. 0.32 0.50
			yes: age as a covariate	no	yes: non-shared environment	biometric model (twin data)	287	0.36 (0.22–0.48)
[46]	human *Homo sapiens*	Southern blot	yes: age as a covariate	no	no	not stated (sibling data)	383 adults /258 sib pairs	0.82 (0.59–1.05)
[47]	human *Homo sapiens*	qPCR	yes: age-adjusted telomere length	yes: adjusted for parental age at birth	no	father–offspring father–son father–daughter mother– offspring mother–son mother–daughter	42 20 22 41 18 23	1.13 1.08 1.21 n.s. n.s. n.s.
[48]	human *Homo sapiens*	Southern blot	yes: age as a covariate	no	yes: shared familial environment	structural equation model (twin data)	1025	0.36 (0.18–0.48)
[49]	human *Homo sapiens*	qPCR	yes: age-adjusted telomere length	no	no	father–son father–daughter mother–son mother–daughter	62 102 63 105	1.12 0.86 n.s. n.s.
			yes: age as covariate	no	yes: environmental risk factors (e.g. age and sex)	‘animal’ model	907	0.44 (0.32–0.56)
[50]	human *Homo sapiens*	Southern blot	yes: age-adjusted telomere length	no	yes: shared and individual environment	linear mixed model (twin data)	306	n.s.
[51]	human *Homo sapiens*	Southern blot	no	no	no	linear mixed model (twin data)	175	0.56 (0.42–0.67)
[52]	human *Homo sapiens*	qPCR	no	no	no	parent–offspring (centenarian parents)	86	0.86
[53]	human *Homo sapiens*	qPCR	yes: age-adjusted telomere length	no	no	father–son father–daughter mother–son mother–daughter grandparent–grandchild	51 47 57 72 85	0.93 0.97 n.s. 0.59 1.09
[54]	human *Homo sapiens*	qPCR	yes: age-adjusted telomere length	yes: adjusted for parental age	no	parent–offspring: leukocytes CD34 + cells mononuclear cells buccal cells	not stated	0.90 0.79 1.09 0.74
[55]	human *Homo sapiens*	qPCR	yes: age as covariate	no	yes: cohort as covariate	parent–offspring	41	1.32
[56]	human *Homo sapiens*	qPCR	yes: age-adjusted telomere length	no	no	siblings MZ twins DZ twins father–son father–daughter mother–son mother–daughter	1553 2534 2172 791 882 850 1005	0.98 0.69 1.00 0.68 0.66 0.84 0.84
			yes: age as covariate	no	no	meta-analysis (based on estimates from 6 ‘animal’ models)	19 713	0.70 (0.64–0.76)
[57]	human *Homo sapiens*	qPCR	yes: age as covariate	no	yes: education, site, smoking, alcohol consumption and marital status as covariates	animal model: all data males females	4289 1927 2362	0.54 (0.47–0.61) 0.60 (0.47–0.72) 0.52 (0.42–0.62)
[58]	human *Homo sapiens*	qPCR	yes: age as covariate	no	no	‘animal’ model	3587	0.56 (0.50–0.61)
[59]	human *Homo sapiens*	qPCR	yes: age as covariate	no	no	‘animal’ model: all data excluding haematological malignancies	1079 949	0.63 (0.35–0.90) 0.76 (0.46–1.05)
[60]	human *Homo sapiens*	qPCR	yes: gestational age as covariate	yes: maternal age as covariate	yes: shared and individual environment	structural equation model (twin data)	162	0.13 (0.00–0.69)
[61]	human *Homo sapiens*	qPCR	no	yes: controlled for parental age	yes: controlled for education	father–ADHD offspring mother–ADHD offspring	37 57	1.26 (0.70–1.82) 1.12 (0.76–1.48)
[62]	human *Homo sapiens*	Southern blot	yes: age as covariate	no	yes: shared and individual environment	linear mixed model (twin data)	652	0.64 (0.39–0.83)
[63]	human *Homo sapiens*	qPCR	yes: age as covariate	no	yes: education, site, smoking, alcohol consumption and marital status as covariates	animal model: all data fathers & offspring mothers & offspring fathers & male offspring fathers & female offspring mothers & male offspring mothers & female offspring	3040 3404 3568 1855 2147 2016 2311	0.54 (0.47–0.61) 0.67 (0.58–0.76) 0.61 (0.52–0.69) 0.65 (0.52–0.79) 0.62 (0.50–0.74) 0.57 (0.44–0.69) 0.53 (0.42–0.63)
[64]	human *Homo sapiens*	not stated	yes: age-adjusted telomere length	no	no	MZ twins	210	0.88
[65]	human *Homo sapiens*	qPCR	yes: age as covariate	no	yes: education as covariate	‘animal’ model with SNP-based relatedness	3290	0.28 (0.03–0.53)
[66]	human *Homo sapiens*	qPCR	yes: age-adjusted telomere length	no	no	pairwise familial correlations	1780	0.63
[67]	human *Homo sapiens*	Southern blot	no: babies < 2 week old, and mothers with babies with Down syndrome or control babies were aged matched	no	no	mother–offspring with Down syndrome (MI) mother–offspring with Down syndrome (MII) mother–offspring (control group)	106 64 186	−0.12 (−0.15 to −0.09) −0.13 (−0.16 to −0.11) −0.16 (−0.20 to −0.11)
[68]	kakapo, *Strigops habroptilus*	Southern blot	no: but no TL–age correlation	no	no	mother–offspring mother–daughter mother–son father–offspring father–daughter father–son	29 19 10 26 18 8	0.84 n.s. 1.53 n.s. n.s. n.s.
[69]	sand lizard, *Lacerta agilis*	Southern blot	yes: residuals from TL–age regression	no	no	mother–daughter father–son	55 40	0.52 (0.09–0.95) 1.23 (0.80–1.66)
[70]	collared flycatcher, *Ficedula albicollis*	qPCR	yes: nestlings sampled at day 12	no	yes: cross-foster brood triplet	‘animal’ model with cross-fostered siblings	359	0.09 (−0.04–0.15)
[71]	King penguin, *Aptenodytes patagonicus*	qPCR	no: chicks measured at day 10, but parents during brooding	no	no	mid-parent–offspring mother–offspring	53 53	0.2 (−0.02–0.42) 0.2 (0.01–0.39)
[31]	great reed warbler, *Acrocephalus arundinaceus*	qPCR	yes: TL measured at days 8–10	no	no	mother–mid-offspring mother–mid-daughter mother–mid-son father–mid-offspring father–mid-daughter father–mid-son	17 17 17 19 19 19	1.08 1.12 (0.34–1.90) 1.38 (0.40–2.36) n.s. n.s. n.s.
				yes: maternal age (paternal age had no significant effect)	yes: maternal identity^b^	‘animal’ model	193	0.48 (0.24–0.72)
[72]	zebra finch, *Taeniopygia guttata*	TRF	yes: log(age) as covariate	no	yes: family, maternal or paternal identity	full-sibling maternal half-sibling paternal half-sibling	42 8 18	1.18 (0.46–1.90) 1.35 (−1.04–3.74) 0.93 (−0.27–2.13)
					no^c^	‘animal’ model with cross-fostered siblings	125	0.999 (0.87–1.00)
[73]	white-throated dipper, *Cinclus cinclus*	qPCR	yes: nestling age at sampling as covariate	no	no	mother–mid-offspring father–mid-offspring mother–mid-offspring father–mid-offspring	59 59 59 59	0.44 (0.048–0.83) 0.08 (−0.35–0.51) 0.44 (0.048–0.83) 0.08 (−0.35–0.51)
					yes: nest and year of birth	‘animal’ model	177	n.s. [0.038 (−0.10–0.17)]

^a^*N*: number of relative pairs in regressions or phenotyped individuals in ‘animal’ models. 95% CI are stated for studies providing these data, or where s.e. was provided this was multiplied by 1.96 to estimate the CI.

^b^‘Animal’ model including brood identity did not converge.

^c^‘Animal’ models including parental effects did not converge.

^d^Heritability estimates calculated from parent–offspring regressions (as the slope divided by the coefficient of relatedness) can mathematically be greater than one, whereas heritability is bound between 0 and 1.

## 4. Telomeres are not a fixed trait, but change with age

**Figure 3.**
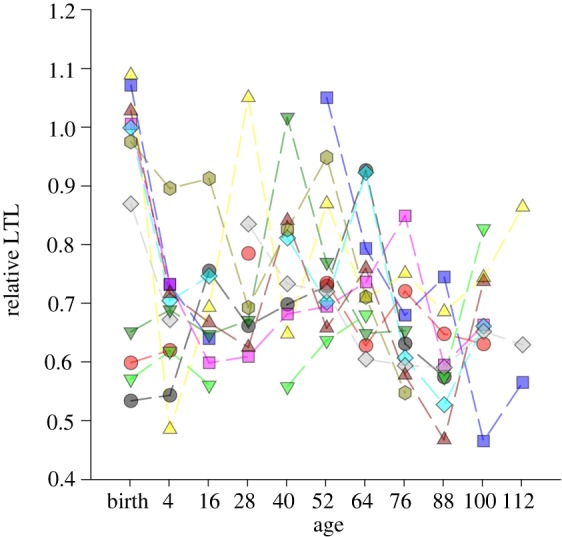
Leukocyte telomere length (LTL) dynamics for 11 female Soay sheep, *Ovis aries*, measured twice as lambs and at least six further times thereafter during their lives. Each colour and symbol combination represents a different individual. Adapted from [20].

Telomeres of somatic cells shorten with age in most organisms, with the amount of shortening depending, to a considerable extent, on the stress experienced as the individual interacts with its environment [7,27]. Consequently, even if the environment remains constant the relative influence of the environment on an individual's telomere length will increase with age. Furthermore, rates of telomere attrition may differ across an individual's lifetime, e.g. attrition is normally much greater during development [30,37]. Telomere attrition may also vary considerably across time because of specific life-history events (e.g. reproduction [74]) or environmental experiences (e.g. infection [31]). Studies that have measured longitudinal changes in telomere length generally find a log-linear relationship with age, but with considerable fluctuations within individuals (figure 3), including evidence that an individual's telomere length may increase over certain periods [20,30].

Interactions with the (changing) environment will also mean that the pattern of change will be volatile, difficult to predict and may vary markedly between individuals. Such age-related variation in telomere length must be carefully controlled for—in both the focal individual and the parents—if we want to gain accurate measures of the heritability of telomere length or dynamics. However, few studies have measured telomeres at the same point in life in both offspring and parents [31]. Given that environmental effects are especially pronounced during early development, including prior to birth/hatching [75,76], having measures from the zygote just after conception would minimize environmental effects, but this is clearly not very feasible.

Ironically, even measuring the telomeres of newly conceived zygotes would not eliminate all age-related effects. Although the initial length of an individual's telomeres may largely be determined by genetic factors within the individual, parental effects may also play a role and cause trans-generational effects, e.g. if the age of a parent influences offspring telomere length. The effects of paternal age at conception (PAC) on offspring telomere length have been widely reported in humans, with older fathers having offspring with longer telomeres [56]. The evidence suggests that this is due to sperm from older males having longer telomeres, either because of age-related selection of germline stem cells or the elongation of telomeres because of telomerase activity [77]. Thus, zygotes produced by such sperm from older males have longer telomeres. In other vertebrates, the evidence of PAC effects are mixed, with negative effects detected [69] or not [31], along with positive maternal age effects [31]. However, many studies are cross-sectional, so selective disappearance may result in, or strengthen, positive correlations. Additionally, the environment experienced by the parent may impact on parental age patterns or influence initial offspring telomere length irrespective of parental age [38]. If such non-genetic trans-generational effects do influence initial zygote length they will also confound our measures of telomere heritability unless controlled for.

## 5. Quantitative genetic techniques

Individual variation in telomere length can be decomposed into the relative variance due to genetic and environmental factors using quantitative genetic techniques [34,35]. The extent to which phenotypes are genetically determined (i.e. heritable) is analysed by examining the phenotypic similarity between relatives. Currently, the most commonly applied technique to estimate the heritability of telomere length is univariate regression analysis [72], but key assumptions of this technique are often overlooked. For example, parent–offspring regressions do not always account for repeated measures of parents that have multiple offspring. Most importantly, relatives often live in more similar environments than non-relatives and share common environmental effects, which can result in relatives having similar telomere lengths for reasons other than genetic effects [1]. Unless this environmental similarity is partitioned from the genetic effects, this will severely confound heritability estimates and lead to overestimations. Cross-fostering is a useful tool that facilitates the separation of genetic from environmental effects. In particular, cross-fostering enables better resolution of additive genetic effects, as it allows the separation (via modelling) of the foster (early-life environmental) and natal (genetic and pre-fostering environmental effects) in addition to additive genetic effect.

More sophisticated mixed model techniques allow separation of phenotypic variance into individual and residual variance components (which allows calculation of repeatability [78]), when multiple telomere measures from the same individuals are available (figure 1*b*, Model 3). These mixed models can then be extended into ‘animal’ models that use family trees with different types of relatives (grandparent–grandoffspring, aunts–nieces, etc.) to separate the individual variance into genetic and permanent environment (environmental effects that are consistent over an individual's repeated measures) components [36] (figure 1*b*, Model 4). These variance components can then be used to calculate narrow-sense heritability (*h*^2^; the proportion of phenotypic variance due to additive genetic effects). In the simplest form, *h*^2^ = *V*_A_/*V*_P_, where *V*_A_ is variance due to additive genetic effects and *V*_P_ is phenotypic variance (*V*_P_ = *V*_A_ + *V*_PE_ + *V*_R_, where *V*_PE_ is permanent environment variance and *V*_R_ is the residual variance that is usually a result of other environmental effects [34,35] (figure 1*b*, Model 4). If permanent environment effects are not incorporated this will result in inflated heritability estimates [1]. Shared environmental effects, such as maternal, paternal, nest, cohort and spatial effects can also be confounded with other variance components (figure 1*b*, Model 5), such as *V*_A_ if they are not specified separately [1,36].

Quantitative genetic ‘animal’ models can also be extended to calculate G × E or Genotype by Age (G × Age) effects, when additive genetic effects vary across environments or with age. A random regression ‘animal’ model [79] allows the slope of a genotype to vary across an axis of environment or age. For example, this would, when applied to repeated telomere length data over the lifetimes of individuals, allow the testing of whether telomere shortening-rates differ according to genotypes. G × E or G × Age models require very large sample sizes, but have been run successfully on traits in natural populations [25].

Quantitative genetic techniques primarily assume additive effects of many genes, however, dominance effects such as inbreeding can impact telomere length, as observed in the Seychelles warbler [32]. If dominant effects are not modelled, they can be confounded in other variance components, e.g. increasing both *V*_A_ and *V*_R_ [80]. However, very large sample sizes, 20× more than that required for estimating *V*_A_, are required to estimate dominance variances accurately [80,81]. Estimation of dominance variance has been undertaken by animal/plant breeders, but is also theoretically achievable in natural populations [82].

## 6. Past studies

Here we review published studies on the heritability of telomere variation in laboratory and natural vertebrate populations, including humans (table 1). Unravelling the role of genetic and environmental effects on similarity in telomere variation in natural populations is difficult, but potentially most important from an evolutionary and ecological perspective. No clear overall patterns are yet emerging. Some studies have reported significantly higher paternal than maternal heritability of telomere length [49,53,69], the opposite effect [56], X-linked [44], no effect [63,72,73], or have found heritability from mother-offspring but not father-offspring regressions [31,68,71], or the opposite effect [47], but have not tested for a significant difference in slopes. The only clear pattern is that there is extensive variation (from 0 to 1!) in the estimates of telomere length heritability (table 1). Indeed, even within a single species (i.e. humans, the species in which most studies have been undertaken) heritability estimates vary massively. However very few, if any, of the studies undertaken so far are without considerable limitations or problems.

The variation in estimates of telomere length heritability may, to some extent, be attributed to methodological issues. First, many studies apply basic regression analyses [72], with all of the problems that this entails, such as confounding genetic and environmental effects. Using twin studies, as often undertaken in humans (table 1) does not fully resolve this problem (see section 7). Secondly, despite the fact that telomeres change with age (see section 4) studies normally sample parents and offspring at different ages (i.e. as adults and juveniles, respectively), especially in long-lived organisms. Many human studies have attempted to statistically control for parent/offspring age, by including age as a covariate or correcting telomere length for age (but this does not allow for the expected nonlinear relationship), and very few also control for parental age at conception (but see [31,47,54,60,61]). In other species, age has also rarely been fully controlled for (table 1). Clearly, it would be better to sample parents and offspring at the same age (see section 4). One excellent study on great reed warblers that did sample both parents and offspring at the same age (8–10 days) found moderate heritability [31]. All the other non-human studies had shortcomings as they did not control for offspring and parental age at sampling, used an age-adjusted telomere length and/or only included offspring age, and sometimes parental age, as a covariate in the model (table 1). Thirdly, despite the fact that environmental effects can vary spatially and temporally (see section three), few studies have accounted for this in their analyses (table 1). The one study undertaken under controlled laboratory conditions (thus reducing environmental variation) also reduced shared environmental effects through cross-fostering and reported a heritability value of 1 [72]. The very high heritability estimate in this case is perhaps not surprising, because once environmental variation is minimized the rest of the variation must be due to genetic effects. However, heritability estimates taken under such conditions are of minimal use to biologists wanting to understand the evolutionary and ecological significance of telomere variation. Spatio-temporal variation in environmental effects may be particularly important when estimating heritability in species either with long generation times, living in rapidly changing environments, or that exist across a range of different environments. In humans, it is interesting to consider how much of the variation in heritability estimates may be due to differences in the environments in which the subjects of each study lived. In the case of parent/offspring studies in humans, how much the environment changed between generations may also be very important. One would not expect much correlation between the telomere length of offspring and parents if the two generations developed under very different environmental conditions, even if sampled at the same age. Another important issue in estimating telomere heritability is that ‘animal’ models are required to separate genetic and environmental effects, and these models require large sample sizes. For example, the seven human studies that applied ‘animal’ models had sample sizes greater than 900 (table 1), whereas two of the four studies in non-human populations that have used ‘animal’ models had models that did not converge when environmental effects were included (table 1); with less than 230 phenotyped individuals and the one study that attempted more sophisticated sex-linkage models was severely underpowered [73]. The exact sample size required to separate environmental from genetic effects depends on data structure, but samples of an order of magnitude higher than the norm in previous non-human studies are probably required for meaningful results. Fourthly, G × E effects may occur and these have not yet been tested for in any quantitative genetic analyses of telomere length in vertebrate studies. Fifthly, the technique used to assay telomere length may, or may not, include interstitial telomeres (table 1), and it is not known how this affects heritability estimates [72].

The limitations identified in these studies of telomere length will also apply to studies on the heritability of telomere shortening. Currently, the heritability of telomere attrition has only been investigated with twin data [62,84], which has methodological problems [85] (see section 7). Studies that take a G × E approach are desperately needed to improve our understanding of the evolution of telomere dynamics.

## 7. Potential solutions and their problems

First, do not use simple parent–offspring or sibling regressions when relatives share environmental components as this will inflate heritability estimates [1]. Even in studies comparing monozygotic and dizygotic twins this can be a problem; the similarity difference between these types of twins is assumed to be attributed to greater genetic similarity of monozygotic twins, however, the environmental similarity of monozygotic and dizygotic twins is rarely the same [85]. Rather, studies carefully measuring both relatedness and environmental similarity across individuals, and then using analytical methods such as ‘animal’ models [36] to separate genetic from environmental variance components, should be used.

Second, control for the age at which all individuals are sampled (accounting for any measurement effects due to length of storage, extraction or batch differences). Sampling both offspring and adults at the same age will standardize the environmental exposure each party has endured prior to sampling, though clearly the environmental impact could differ for each individual. Sampling all parties as young as possible appears attractive as it should minimize environmental influence. However, there may be situations where it is of interest to measure heritability at different time points. For example, heritability estimates calculated from samples taken from offspring and parents when they are both a given adult age will include more information about the genetic basis of resistance to telomere attrition and/or telomere restoration mechanisms, not just initial telomere length. Measuring how additive genetic variance changes with age is also required to improve understanding of the evolution of senescence [86]. When to sample, therefore, depends very much on what you want to understand.

Third, both physical and analytical means can be used to separate environmental and genetic effects. Cross-fostering can create situations where relatives are raised in different environments. However, individuals will still experience similar environments from conception until cross-fostering, for example, any maternal effects in terms of prenatal investment [1]. Fortunately, in many species a female's offspring may be fertilized by multiple males, often within a single litter/clutch [87]. Even within socially monogamous species extra-pair paternity often occurs [88]. These cases result in offspring from multiple paternal origins, but with the same mother, living under the same conditions and in offspring from the same father but with different mothers, being raised in different environments. These differences provide statistical power to separate genetic and environmental variance components. Indeed, systems where polyandry is frequent and can be combined with cross-fostering at an early stage would provide the most power to resolve effects. Though clearly in some species, e.g. humans, deliberate cross-fostering is not an option!

Although ‘animal’ models provide analytical solutions to separate variance components, as already stated they do require large sample sizes [89]. The exact sample size required to have sufficient power to detect a given heritability value depends on many factors, including: the number of related individuals in the pedigree, the number of pedigreed individuals that have been phenotyped, the pedigree structure (e.g. pedigree depth, completeness and family sizes) and the confidence with which relationships have been assigned in the pedigree (e.g. have extra-pair paternities been accounted for? [90]). It is, therefore, not possible to provide universal guidelines on the sample size required to detect heritability. Accurate heritability estimates may, in certain cases, be estimable from a hundred individuals [2], however, in most cases, samples of an order of magnitude higher than this—and than used in previous non-human studies—are probably required. Importantly, studies must provide sample sizes of the number of phenotyped individuals in the pedigree, the number of phenotyped individuals with repeat measures and the pruned pedigree size (i.e. where uninformative individuals are removed) to allow basic comparisons between studies. Additionally, given that datasets are so variable, the statistical power with which each dataset can detect heritability of a given value [91] should always be reported. Sensitivity analyses can also be run to investigate the impact of particular errors on parameter estimates [89].

Fourthly, to detect G × E or G × Age effects, function-valued trait approaches [92] can be used, or random regression ‘animal’ models (where the slope of the genotype is allowed to vary over environments [41]). Models need to be built in a hierarchical process, first testing for individual×environment effects and then comparing with a G × E/G × Age model. However, detecting G × E/G × Age requires data from individuals experiencing variable or different environments over their lifetimes and very large sample sizes. Currently, no published study has tested for G × E effects of telomere length, although G × E and G × Age effects have been estimated for other traits in natural vertebrate populations (e.g. [25]).

Finally, a general problem with quantitative genetic studies is a lack of consistency in the way in which parameter estimates are presented [25]. Variance components need to be presented in standard hierarchical models [41] to illustrate the way in which variance components are confounded, depending on model specification (figure 1*b*). This then facilitates cross-species comparisons. Importantly, all variance estimates, and the confidence intervals around these, should be reported from all of the models run, so that shifts in variance components can be easily compared between models. Furthermore, the inclusion of covariates can alter heritability estimates from ‘animal’ models, so covariates must be clearly specified [93]. Additionally, measurement error, such as observer bias or batch effect, may need to be controlled for. For example, when measuring telomere lengths using quantitative PCR (qPCR) or telomere restriction fragment (TRF) methods plate or gel effects, respectively, can potentially affect variance components and need to be properly modelled. Once the appropriate models are run, providing a standardized estimate of the additive genetic variance, through a measure of evolvability (*I*_A_), *V*_A_/*μ*^2^, where *V*_A_ is scaled by the population mean telomere length *μ*, facilitates comparison across populations with different mean telomere lengths, given variation scales with the mean [83].

## 8. Conclusion

Having accurate estimates of the heritability of telomere variation in natural populations is fundamental to our understanding of the evolutionary and ecological importance of telomeres. Environmental effects on telomere length can be complex and extensive and contribute significantly to the lack of clarity and consensus from studies that have attempted to estimate the heritability of telomeres so far. Only by fully understanding the nature and timing of environmental effects, and then controlling for them, can we get accurate and meaningful measures of the heritability of telomere length. While there are many difficulties associated with doing this in natural populations there are potential methodological and analytical solutions. To be successful, future studies need to be carefully designed in terms of sampling individuals and ensuring sufficient power to use these solutions. Finally, future studies must present their sample sizes and results in a standard way to facilitate meta-analyses so we can gain a more universal understanding of the causes and consequences of telomere variation.

## Data accessibility

This article has no data.
